# Revealing the Nature of Chronic Obstructive Pulmonary Disease Using Self-tracking and Analysis of Contact Patterns: Longitudinal Study

**DOI:** 10.2196/22567

**Published:** 2021-10-19

**Authors:** Klaus Phanareth, Astrid Laura Dam, Martin A B C Hansen, Signe Lindskrog, Søren Vingtoft, Lars Kayser

**Affiliations:** 1 Epital Health Gentofte Denmark; 2 Department of Public Health University of Copenhagen Copenhagen Denmark; 3 Region Zealand Sorø Denmark

**Keywords:** COPD, exacerbations, patient-reported outcomes, Epital Care Model, early interventions

## Abstract

**Background:**

Chronic obstructive pulmonary disease (COPD) is the fourth leading cause of death and is characterized by a progressive loss of pulmonary function over time with intermittent episodes of exacerbations. Rapid and proactive interventions may reduce the burden of the condition for the patients. Telehealth solutions involving self-tracking of vital parameters such as pulmonary function, oxygen saturation, heart rate, and temperature with synchronous communication of health data may become a powerful solution as they enable health care professionals to react with a proactive and adequate response. We have taken this idea to the next level in the Epital Care Model and organized a person-centered technology-assisted ecosystem to provide health services to COPD patients.

**Objective:**

The objective is to reveal the nature of COPD by combining technology with a person-centered design aimed to benefit from interactions based on patient-reported outcome data and to assess the needed kind of contacts to best treat exacerbations. We wanted to know the following: (1) What are the incidences of mild, moderate, and severe exacerbations in a mixed population of COPD patients? (2) What are the courses of mild, moderate, and severe exacerbations? And (3) How is the activity and pattern of contacts with health professionals related to the participant conditions?

**Methods:**

Participants were recruited by convenience sampling from November 2013 to December 2015. The participants’ sex, age, forced expiratory volume during the first second, pulse rate, and oxygen saturation were registered at entry. During the study, we registered number of days, number of exacerbations, and number of contact notes coded into care and treatment notes. Each participant was classified according to GOLD I-IV and risk factor group A-D. Participants reported their clinical status using a tablet by answering 4 questions and sending 3 semiautomated measurements.

**Results:**

Of the 87 participants, 11 were in risk factor group A, 24 in B, 13 in C, and 39 in D. The number of observed days was 31,801 days with 12,470 measurements, 1397 care notes, and 1704 treatment notes. A total of 254 exacerbations were treated and only 18 caused hospitalization. Those in risk factor group D had the highest number of hospitalizations (16), exacerbations (151), and contacts (1910). The initial contacts during the first month declined within 3 months to one-third for care contacts and one-half for treatment contacts and reached a plateau after 4 months.

**Conclusions:**

The majority of COPD patients in risk factor group D can be managed virtually, and only 13% of those with severe exacerbations required hospitalization. Contact to the health care professionals decreases markedly within the first months after enrollment. These results provide a new and detailed insight into the course of COPD. We propose a resilience index for virtual clinical management making it easier to compare results across settings.

## Introduction

Chronic disease poses one of the biggest challenges facing Western health care systems, and with its increasing prevalence in the population, it is a major driver of health care utilization and the leading cause of death [1]. Chronic obstructive pulmonary disease (COPD) is one of the chronic diseases that weighs heavily in this scenario. Currently the fourth leading cause of death in the world, COPD is projected to be the third leading cause of death by 2020 [2] with an increasing socioeconomic burden and growing strain on health care budgets [3]. COPD is characterized by a progressive loss of pulmonary function over time with intermittent episodes of exacerbations as part of its natural history [2,4]. The long-term course of the disease depends on how well individuals can understand, manage, and act upon risk factors such as smoking, nutritional state, exercise activity, and compliance to medical treatment plans [5]. In the short-term, the prevention and treatment of exacerbations are key elements in the management of COPD according to the Global Initiative on Chronic Obstructive Lung Disease (GOLD) as the number of exacerbation is closely associated with an increased rate of hospitalization, increased mortality, decline in lung function, increased use of health care resources, and decrease in quality of life [2,4]. According to the GOLD guidelines, COPD exacerbation is defined as “an acute worsening of respiratory symptoms that results in additional therapy.” These events are classified as mild, moderate, or severe, of which the first 2 categories are characterized by the type of medication given and the latter characterized by the patient’s need for hospitalization or visits to the emergency room [2].

Early detection of exacerbations and prompt interventions with antibiotics and corticosteroids can reduce hospital admissions, decrease use of health care services, and increase and improve quality of life [6,7]. Sixteen years ago, Wilkinson et al [8] showed that early recognition of exacerbations using symptom-based diaries completed by the patient followed by prompt treatment by a physician is beneficial to the recovery of the exacerbation and can improve the burden of exacerbation-related morbidity and mortality. The authors concluded a need for initiatives to encourage reporting of early presentation of exacerbations and enhanced symptom recognition tools that are easy to use and promptly managed by health professionals.

Telehealth solutions involving self-tracking of vital parameters such as pulmonary function, oxygen saturation, heart rate, and temperature with synchronous communication of health data may become a powerful solution in this context as it enables health care professionals to react with a proactive and adequate response. Over the last decade, data from several randomized controlled trials examining the effect of telehealth and how it may assist self-monitoring and management in COPD have been reported [9-12]. Some studies suggest a potential beneficial effect with a reduction of exacerbations [13], acute hospital admissions, and mortality [14] and increased health-related quality of life [15] whereas others have not been able to verify these results [16-18]. In COPD, no studies have yet demonstrated conclusively that telehealth is neither superior nor inferior to usual care; it is noteworthy that telehealth interventions have not caused any harm to COPD patients and the technology has been welcomed by the users [10,19,20].

One of the fundamental elements of a person-centered and value-based health care provision of services is the inclusion of the patients’ self-reported data including their experiences as patients. These data are often termed patient-reported outcomes (PROs) [21]. PROs are a natural component of telehealth as they can be used to report the condition dynamically over distance to service providers. PROs may also engage the patients and increase self-management due to a better insight into their own condition [21]. In the context of COPD, PROs are a key element for patients to communicate with their health providers, enabling them to understand their condition and proactively act upon potential deteriorations [22].

Telehealth and PROs may contribute to the fulfillment of the World Health Organization framework for integrated people-centered health services as they facilitate the engagement and empowerment of patients and the reorientation of how health care can be delivered [23].

In an attempt to demonstrate how these ideas and potentials can be realized, we have developed a new person-centered service model: the Epital Care Model (ECM). We have tested the ECM in a living lab [24] and evaluated potential candidates for PROs gathered by psychometric validated instruments focusing on self-management, health literacy, and physical and mental well-being as well as clinical parameters [25]. In this study, we report on the second part, in which we, based on a newly developed algorithm and setting (to be reported elsewhere), are able to evaluate how PROs relating to the clinical condition can be used to understand the actual needs of the patient and needs of resources required from the ECM living lab to help the patient return to their habitual condition.

For this purpose, we have followed the self-tracking activities of 87 patients with COPD and the corresponding standardized responses from health care professionals when responding to signs of deteriorations of the patients’ clinical condition or if the patients need support to manage their condition.

To understand the activities and required resources, we have addressed the following research questions:

- What is the incidence of mild, moderate, and severe exacerbations in a mixed population of COPD patients?
- What is the course of the mild, moderate, and severe exacerbations?
- How is the activity and pattern of contacts with health professionals related to the participant condition?

## Methods

The setup and initial data of this study has been described previously by Phanareth [24]. In short, the data for this study was collected as part of the Epital living lab project which was established in a Danish municipality in 2012 [24].

### Setting

The Epital Care Model is a model designed to help organize health care services both virtually and physically with a personalized health-centered approach and with the objective of enhancing empowerment and self-management. Services and interactions are organized according to 6 levels that can be divided into three 3 settings depending on the resources allocated. In the ECM1 level, participants live an independent and active life with their condition supported by technology. When participants need medical support in relation to their condition, they are classified as being at the ECM2 level. Both ECM1 and ECM2 interactions and services can be provided at any location and are performed virtually (setting 1). If the participant’s condition deteriorates and a physical visit is necessary, nurses will go to the participant’s physical location and assess the condition thereby placing the participant at the ECM3 level. If regular follow-ups by nurses and medical doctors are needed, this will be offered as a patient at home service termed “outmission” at the ECM4 level (setting 2). The outmission is equivalent to services offered when admitted to a hospital but in this case, they are provided at home by a virtual medical doctor (eDoctor) and a nurse (setting 3). The eDoctor performs virtual ward rounds but the patients can receive visits from community nurses at the participant’s physical location if necessary. At the ECM5 and ECM6 levels, the participant is now considered a patient and is referred to a community or hospital bed depending on the need of care and monitoring.

### Population

The study period took place from November 1, 2013, to December 31, 2015, and in this period, we consecutively enrolled participants recruited by a snowball-like method based on convenience sampling. This resulted in a random sampling over seasons and an uneven period for observations defined by either the time from enrollment to the planned end of the study or a period defined as the time from enrollment to the participant’s death or choice to withdraw from the study.

A total of 93 participants with COPD were enrolled into the ECM living lab, of these 27 were transferred directly from the prior pilot study and 66 were recruited throughout the period of the study. For this analysis, 5 were excluded due to not having provided any measurements after the inclusion and one due to insufficient inclusion data, resulting in a total of 87 participants.

During the study, 29 patients dropped out: 9 of whom because they died, 8 for reasons not associated to the tracked exacerbations, and 12 for other reasons. In addition, 5% (4/87) of participants dropped out within 14 days, but data from these participants are included as an objective of this study was to assess the overall activity and event of the participants.

At entry, all participants were registered with baseline data including age, sex, heart rate, oxygen saturation, forced expiratory volume for 1 second (FEV_1_), and a clinical examination [25]. Based on these data, participants were classified according to the previous GOLD guidelines from 2011 [26], as the study was planned in 2013. They were classified according to the air flow limitation severity in COPD (GOLD classes I-IV) and their risk factor group (GOLD risk factor groups A-D) assessed by the number of exacerbations per year and their Medical Research Council breathless test score. We recognize that these recommendations have changed in new edition of the GOLD guidelines and our data may therefore not be directly comparable to newer data sets [26].

### Context and Data Collection

The ECM is a unique, personalized health-centered living lab project that consists of an environment and service ecosystem where it is possible to investigate everyday fluctuations when living with COPD. Simultaneously, the ECM makes it possible to estimate how many resources are needed to meet the needs of the participants both when the participants live an active and independent life in ECM1 and when their condition deteriorates and they are allocated resources as described in ECM2 to ECM6.

The ECM living lab provides the participants with 24/7 access to assistance from the response and coordination center (RCC) manned by nurses supported by eDoctors. The self-tracking activities are completely regulated by the participants, driven by either curiosity or needs in relation to the ongoing changes of their condition. As the participants register their data, they are simultaneously reported to the RCC. The participants are informed that their data will be visible for the RCC staff, they can expect the RCC to respond if their condition deteriorates, and self-tracking is digital assistance helping them to manage their condition. The participants are also able to contact the RCC through their tablet using video or voice communication if they feel insecure or have any questions [24].

A third kind of communication may be initiated by the RCC staff: when they register a deterioration, they will contact the participant to investigate whether there is any need of assistance. For the participants, this data-driven approach should result in a structured and less intrusive way to live with their COPD as they only need to report their PRO data when they feel like it and only contact the RCC if they need advice, care, or treatment.

The information technology (IT) support for processes and activities in the ECM living lab constitutes 2 IT systems specially tailored for this purpose [24]: a citizen-oriented monitoring system (Appinux Care, Appinux A/S) and a medical documentation system (EpiProcess, OCEAN Process A/S).

The COPD monitoring module in the Appinux platform allows participants to deliver real-time data to health care providers. Participants used a Samsung tablet with an app to report their data used to assess their condition. The participants themselves reported a measured body temperature, increased breathlessness, increased coughing, and changed color of sputum. These data were reported together with a guided semiautomatic measurement of heart rate, oxygen saturation, and FEV_1_. The initial assessment of the data is performed by the system, and an algorithm presents this assessment to the participant as a color (red, yellow, green) along with advice on the appropriate action to take.

Based on these data, the health care professionals can, together with the participant, make informed decisions on how to best manage the change in condition. This will be based on the previous measures evaluated with graphs, including plotted trends, which enable the health professionals to better predict the course of the participant. All participants were equipped with an acute medicine box at home, to be used only when exacerbations occurred to avoid delays in the initiation of a medical treatment. The content was prescribed by the eDoctor, provided by a local pharmacy, and used only in agreement with the RCC staff. The acute medicine box contained bronchodilators (short-acting β_2_-agonist and long-acting β_2_-agonist), inhaled corticosteroids, prednisolone tablets, and broad-spectrum antibiotic tablets.

The medical treatment was standardized to match 3 categories of exacerbations (mild, moderate, and severe) using a stepwise approach with increasing intensity of the medication to match the severity (Table 1). The initiation of medical treatment is based on symptoms and the algorithm system using the PRO data. Based on these and the history, the participant was treated with drugs according to Table 1. The pattern of used drugs was used afterward to classify the participant’s severity of exacerbation for the purpose of this study.

**Table 1:** Description of severity based on medical prescription.

Corresponding medical treatment	Exacerbation type
Increase of ICS^a^ and LABA^b^ + SABA^c^	Mild
Increase of ICS and LABA + SABA + oral prednisolone^d^	Moderate
Increase of ICS and LABA + SABA + oral prednisolone + antibiotics^e^	Severe

^a^ICS: inhaled corticosteroid.

^b^LABA: long-acting β_2_-agonist.

^c^SABA: short-acting β_2_-agonist.

^d^Course of 37.5 mg daily for 5 to 10 days.

^e^Amoxcillin 500 mg × 3 daily for 10 days.

Our definition of severe exacerbation differs from the one purposed in the GOLD guidelines, as GOLD classifies this as a condition requiring hospitalization or emergency room visits. In our setting, we defined a severe exacerbation as a condition where an increase of inhaled corticosteroid, long-acting β_2_-agonist, short-acting β_2_-agonist, oral prednisolone, and antibiotic was needed (Table 1); this resembled more a moderate exacerbation compared to GOLD. In our case, however, if a severe exacerbation treatment was initiated it triggered a series of standardized health services including a follow-up program with daily virtual ward rounds, close monitoring of the condition, and medical adjustments if needed (ECM3 and ECM4).

We therefore argue that our approach in a combined virtual and physical setting is comparable to the procedures performed during a hospitalization or in the emergency room, and therefore may be covered by the definition of severe exacerbation as proposed in the GOLD guidelines.

### Statistical Analysis

At entry, we registered sex, age, FEV_1_, pulse rate, and oxygen saturation for each participant. During the study, we registered participant observation period, number of exacerbations, and number of contact notes, which were coded into care and treatment notes. Each participant was at entry classified according to the GOLD classes I through IV and placed in a risk factor group A through D. Categorical data were reported as numbers and percentages and continuous data were reported as mean values with standard deviations.

Age at entry; participant days; number of mild, moderate, severe, and total exacerbations; and number of measurements per participant year were compared between the risk factor groups using 1-way analysis of variance. All statistics were performed using Excel (version 2016, Microsoft Corp).

### Ethics and Data Protection

As previously reported, the study was assessed and found not to need specific approval by the regional office of the National Danish Ethics Committee (H-3-2012-FSP31). The program was also registered with the National Danish Data Agency by first the University of Copenhagen (2012-41-0384) and since January 2014 by the municipality of Lyngby-Taarbæk, Denmark (20150910229). All participants were told about the study orally and with written material and informed that they could withdraw from the services at any time. Each participant signed a consent with a copy of this information. All data were handled according to Danish legislation and regulations.

## Results

**Figure 1:**
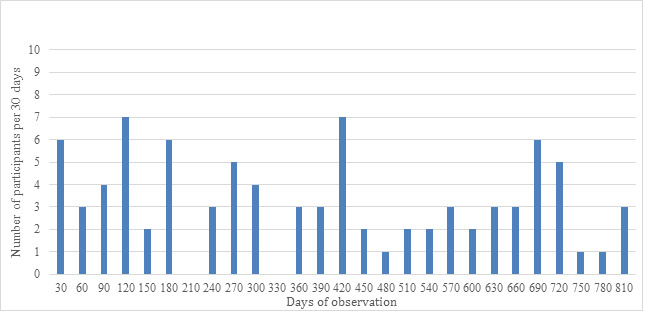
Observation period for the participants.

The study population consisted of 58 females and 29 males. The average age was 73.7 (range 47-91) years. The accumulated number of self-tracked measurements was 12,470. The accumulated number of days with self-tracking was 31,714, and the average of self-tracking days per participant was 368.8 (SD 248.7) with a range from 1 to 791 days per participant. As seen in Figure 1, only 7% (6/87) of the participants dropped out within the first month and 85% (74/87) participated for more than 3 months.

The number of participants classified both according to risk factor groups and severity is shown in Table 2. More than half of all patients were characterized with severe or very severe COPD. The mean FEV_1_ and standard deviation at entry served as baseline for the evaluation of the participants during the observation period (also shown in Table 2).

**Table 2:** Forced expiratory volume during the first second at baseline of the study by participants’ GOLD class and risk group.

GOLD^a^	A: MRC^b^ score <2; 0-1 exacerbations per year, mean (SD)	B: MRC score ≥2; 0-1 exacerbations per year, mean (SD)	C: MRC score 0-1; ≥2 exacerbations per year, mean (SD)	D: MRC score ≥2; ≥2 exacerbations per year, mean (SD)	Total
I: FEV_1_^c^≥80% predicted	—^d^	—	—	1.46, n=1	1
II: 50%≤FEV_1_<80% predicted	1.47 (0.46), n=11	1.52 (0.46), n=23	1.49 (0.05), n=3	0.86 (0.26), n=4	41
III: 30%≤FEV_1_<50% predicted	—	1.28, n=1	1.10 (0.08), n=8	0.74 (0.22), n=16	25
IV: FEV_1_<30% predicted	—	—	0.97 (0.05), n=2	0.67 (0.3), n=18	20
Total	11	24	13	39	87

^a^GOLD: Global Initiative on Chronic Obstructive Lung Disease.

^b^MRC: Medical Research Council.

^c^FEV_1_: Forced expiratory volume during the first second.

^d^Not applicable.

The number of participants were unevenly distributed across the severity classes and risk factor groups (Table 2). Therefore, we adjusted for observation period by normalizing data into participant years or days of observation (Table 3).

The mean number of measurements per participant days did not differ between the risk factor groups, although risk factor group D tended to have higher activity. The numbers are less than one per day and equivalent to a measurement every second to fifth day but with a wide range from 0.01 and up to 3.8 measurements per day.

The number of exacerbations divided into mild, moderate, and severe did not differ significantly among the groups, although the annual rate per patient tended to be higher in risk factor group D with risk factor group B having the second highest mean value. The mean numbers of care notes and treatment notes were similarly higher in risk factor group D, and risk factor group B had the second highest mean of care notes per participant year. The mean for treatment notes per participant year deviated from this pattern as risk factor group C had almost the same number as risk factor group B. This was primarily due to one participant in group C who had 93 treatment notes over a period of 219 days.

A total of 21 of the contact notes were related to other issues than living with COPD, 2 related to treatment of urinary tract infection, 2 to peripheral edema treated with diuretics, and 2 to acute need of hospitalization for other reasons. None of the contact notes was related to treatment or care of other somatic long-term conditions such as ischemic heart failure or diabetes.

In Table 3, the characteristics of exacerbations and contact notes distributed within risk groups are provided.

**Table 3:** Distribution of exacerbations and contacts within risk groups.

Characteristic	Risk factor				
	A (n=11)	B (n=24)	C (n=13)	D (n=39)	Total (n=87)
Participant years, mean (SD)	0.77 (0.57)	1.12 (0.68)	1.16 (0.72)	0.94 (0.66)	1.00 (0.67)
Measurements per participant day, mean (SD)	0.28 (0.18)	0.43 (0.36)	0.35 (0.27)	0.57 (0.55)	0.45 (0.46)
Total number of exacerbations	11	70	23	150	254
Exacerbations/year, mean (SD)	1 (1.49)	2.92 (3.36)	1.77 (2.35)	3.87 (4.76)	2.93 (3.89)
Mild exacerbations	4	29	9	34	76
Mild exacerbation/year (SD)	1.11 (2.91)	1.07 (1.77)	0.52 (1.02)	1.06 (1.71)	0.99 (1.81)
Moderate exacerbations	2	11	7	37	57
Moderate exacerbation/year, mean (SD)	0.18 (0.45)	0.38 (0.97)	0.64 (1.84)	1.06 (1.96)	0.70 (1.61)
Severe exacerbations	5	30	7	79	121
Severe exacerbations/year, mean (SD)	0.28 (0.53)	1.74 (3.50)	0.37 (0.58)	1.85 (2.17)	1.39 (2.41)
Care notes	102	382	124	789	1397
Care notes/year, mean (SD), (n=84)	23.33 (29.06)	21.94 (23.90)	12.33 (11.78)	31.13 (33.55)	24.80 (28.52)
Treatment notes	53	364	166	1121	1704
Treatment notes/year, mean (SD)	5.29 (6.90)	14.20 (22.00)	14.85 (42.40)	29.01 (32.27)	20.28 (30.60)
Total contact notes/year mean (SD)	28.62 (33.41)	37.43 (34.75)	27.18 (50.18)	57.71 (44.74)	45.31 (45.41)

The participants’ total number of contacts were primarily related to ECM2 with the virtual clinical management. Less than 5.36% (165/3081) of the contact notes related to more resource-demanding activities such as evaluation in the participants’ home by a mobile acute team (ECM3 and 4), referral to community bed care (ECM5) or hospitalization (ECM6). As seen with the number of exacerbations, the highest number of contacts were in risk factor group B and D (Table 4). This is partly due to the higher number of participants in these 2 groups, as the adjustment for participant years (Table 3) reduces the difference between group A and C versus group B and D. The differences, particularly in group C, may also be explained by a lower need for contacts not related to treatment as these contacts contribute to the largest difference between group C versus group B and group D (Table 3).

**Table 4:** Number of contact notes (%) within risk factor distributed at ECM levels.

Risk factor	ECM1^a^	ECM2^b^	ECM3^c^	ECM4^d^	ECM5^e^	ECM6^f^	Discharge notes	Total
A	24 (0.8)	129 (4.2)	1 (0.0)	2 (0.1)	0	0	—^g^	156 (5.1)
B	86 (2.8)	629 (20.4)	6 (0.2)	10 (0.3)	3 (0.1)	6 (0.2)	6 (0.2)	746 (24.2)
C	31 (1.0)	242 (7.9)	5 (0.2)	7 (0.2)	0	2 (0.1)	1 (0.0)	288 (9.6)
D	139 (4.5)	1615 (52.4)	35 (1.1)	35 (1.1)	28 (0.9)	25 (0.8)	14 (0.5)	1891 (61.3)
Total	280 (9.1)	2615 (84.9)	47 (1.5)	54 (1.8)	31 (1.0)	33 (1.1)	21 (0.7)	3081 (100)

^a^ECM1: Epital Care Model 1—Digitally facilitated active and independent living.

^b^ECM2: Epital Care Model 2—Virtual clinical management.

^c^ECM3: Epital Care Model 3—Clinical management @home, digitally assisted.

^d^ECM4: Epital Care Model 4—Outmitted @home.

^e^ECM5: Epital Care Model 5—Admitted to local community care facility.

^f^ECM6: Epital Care Model 6—Admitted to hospital.

^g^Not applicable.

**Figure 2:**
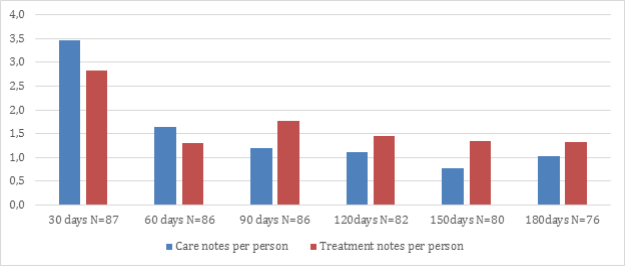
Number of care contact notes and treatment contact notes per person over the first 6 months after enrollment/on-boarding, reported in intervals of 30 days.

The numbers of care and treatment notes are highest in the first month after enrollment in the ECM (Figure 2). The care notes decline over time with a plateau after 3 months at approximately one-third of the initial number of care notes. The number of treatment notes were initially lower than the care notes and reach a plateau after 4 months at approximately half of the initial number of treatment notes. Most of the care and treatment notes (2111/3060, 68.99%) were conducted in the daytime hours between 8:00 AM and 5:00 PM.

Most of the exacerbations were managed virtually in the context of the RCC (ECM2), and 69.4% (84/121) of the severe exacerbations were mitigated virtually (Table 5). Only 21.3% (54/254) of all exacerbations required more intensive diagnostics and follow-up with the majority of these belonging to the severe exacerbations. Only 7.1% (18/254) of the exacerbations required hospitalization and an equivalent number of exacerbations required hospitalization at home (ECM4).

**Table 5:** Number of exacerbations (%) in relation to severity of the exacerbations and the allocated resources (ECM level).

Exacerbation type	ECM2^a^	ECM3^b^	ECM4^c^	ECM5^d^	ECM6^e^	Total
Mild	72 (94.7)	2 (2.6)	2 (2.6)	—^f^	—	76 (100)
Moderate	44 (77.2)	6 (10.5)	5 (8.8)	—	2 (3.5)	57 (100)
Severe	84 (69.4)	4 (3.3)	13 (10.7)	4 (3.3)	16 (13.2)	121 (100)
Total	200 (78.7)	12 (4.7)	20 (7.9)	4 (1.6)	18 (7.1)	254 (100)

^a^ECM2: Epital Care Model 2—Virtual clinical management.

^b^ECM3: Epital Care Model 3—Clinical management @home, digitally assisted.

^c^ECM4: Epital Care Model 4—Outmitted @home.

^d^ECM5: Epital Care Model 5—Admitted to local community care facility.

^e^ECM6: Epital Care Model 6—Admitted to hospital.

^f^Not applicable.

## Discussion

### Principal Findings

This study is, to the best of our knowledge, the first to describe the activities and interactions that take place in an environment based on telehealth and a systematic use of PRO data. An environment was designed to meet the needs of patients and provide an active and independent living in accordance with the person-centered health service model, the ECM [24].

The ECM environment with its service ecosystem offers a unique opportunity to more thoroughly explore the nature of COPD in relation to the severity described as GOLD classes and risk factor groups and the required resources to assist the participants to adequately manage their own condition.

In our study, we detected a total of 254 exacerbations in 87 patients based on PRO measures with a subsequent clinical assessment that resulted in medical treatment corresponding to either mild, moderate, or severe exacerbation (Table 1). Overall, we found 59% (150/254) of the exacerbations occurred in the high-risk group (risk factor group D) with an annual rate of 3.9 exacerbations per participant year, and we found that approximately half of these were categorized as severe exacerbations with 1.86 severe exacerbations per participant year. These results are in the higher end compared to other studies that have also examined annual rates of exacerbations. Comparison of our data with other studies is difficult as it requires a standardization of the severity of the COPD condition and a classification of severity of exacerbations across studies. Our study design builds on the 2011 GOLD guidelines and a pragmatic approach to classification of severity taking the strength of telehealth into consideration, as described in the methods section. Wilkinson et al [8] found, in accordance with our study, a mean of 2.5 exacerbations per patient year, but 40.1% of these were not detected by the clinicians and covered a broad spectrum of patients with respect to severity.

In a review by Seemungal et al [27], the authors found an estimated annual rate of COPD exacerbations as low as 0.5 to a high of 3.5 exacerbations per patient year and hospitalization rates with a range from 0.09 to 2.4 per patient year, the latter being categorized as severe exacerbation according to the GOLD definition [26].

It is surprising that the annual rate of exacerbations in risk factor group C tends to be lower, although not significantly, than in risk factor groups B and D. This may be due to the low number of participants in risk factor group C and is thereby a statistical coincidence and not real finding. In Denmark, patients with COPD will be referred to pulmonary specialists when they move from risk factor group B to C and this may also be a factor that can explain why group C has a lower annual rate of exacerbations than group B.

Our data, which build on a 24/7 self-tracking setup with immediate response from RCC staff, provide us with a detailed insight into the nature of the COPD condition and reports of deteriorations, which may have been missed in other settings. This may explain the higher number of total exacerbations (3.9 per participant year), whereas the number of severe exacerbations is closer to the other studies. Had we used a classification of severity similar to the GOLD guidelines by only including those in need of physical contact to health professionals in a setting equivalent to an emergency room or hospitalization, the number would only have been 37 of the detected 254 exacerbations, which is 31% of the 121 considered to be severe exacerbations based on our criteria or only 15% of all 254 exacerbations. It is also noteworthy that 2 of those initially classified as moderate exacerbations resulted in hospitalization and 16 of the 121 classified as severe exacerbations resulted in hospitalization. If the stricter approach was followed, by only classifying those in ECM3 to ECM6 as severe, 21 of 37 were not hospitalized but mitigated before hospitalization.

The number of exacerbations leading to hospitalizations is similar to the 6.4% found by Wilkinson et al [8]. Whether this is a result of a lack of effect of our setting or due to our inclusion of a higher number of participants in risk factor group D remains to be explored. In a study by Pinnock et al [19] comparing telehealth with conventional care they found an annual rate of number of hospitalizations per patient to be 1.2. This is considerably higher than our 18 hospitalizations out of 87 participants with 0.21 hospitalizations per participant year.

When comparing exacerbation rates, one must therefore be careful and very aware of the lack of consensus of clear definitions of exacerbations, grading systems for the severity of the disease, the high heterogeneity among studies, and the different treatment culture among clinicians which also have an influence.

The higher overall exacerbation rates in this study may be explained by the voluntary self-tracking setup with an easy way to report PRO data, which provides far more data than one would normally get in a traditional health system. This may provide a higher sensitivity for detecting exacerbations but may also decrease the specificity in comparison to traditional systems as the threshold for acting on changes in the pulmonary condition may be lowered. The higher occurrence of exacerbations in our study compared to others, may be explained by the notion that exacerbations go unreported [8,28,29].

Whether a lower threshold for detection and an apparent increased administration of drugs will be reasonable and be considered proactive by being able to prevent a deterioration of the COPD over time by a more aggressive handling of the acute exacerbations needs further studies.

### Association Between Clinical Condition and RCC-Mediated Activities

When organizing a 24/7 service available for all individuals with COPD in a geographical area, it is important to understand whom will need which services and how these needs will evolve over time. As expected, most of the severe exacerbations and hospitalizations were associated with the participants in risk factor group D. This group also had the highest number of contacts, both related to care and treatment. Most of contacts were handled at the virtual clinical management level with 84.1% taken place in relation to ECM2 and with an additional 9.1% only 6% of all contacts were in relation to the more resource demanding activities in ECM3-ECM6.

We find that the activities as estimated by contact notes were more than twice as high during the first month of enrollment into the living lab than after 3 to 4 months. Even the number of treatments notes declined, indicating that the participants learned to better manage their condition or became less distressed. This is in accordance with Kargiannakis et al [30] who, in a service ecosystem comparable to ours, found that the initial contacts per participant dropped from 7 per day to 4 within the first week. The total number of contacts over a period of 42 days with 23 participants were considerable higher with a total of 451 or approximately 14 per participant within the first month [30].

Our results are in accordance with our previous finding that the participants’ emotional distress decreases over time [25]. The reduced number of contacts to the RCC may also reflect the finding that the participants reported a perception of being less active in managing their condition [25]. Based on both the previous findings and the number of contacts, will we hypothesize that the ECM living lab environment and service ecosystem contributes to the participants ability to better manage the COPD and related challenges. It should also be noted that the majority of contacts were in the daytime, which indicates a relatively low need for urgent and often more costly contacts in the evening and at night.

Another important finding is that when an exacerbation is mild when detected, the likelihood of needing physical contact to the health professionals in the course is low (< 5%) and with moderate exacerbations this increases to approximately 23% whereas approximately 31% with severe exacerbations require physical evaluation and approximately half of these needs to be hospitalized.

### Virtual Clinical Management Resilience Index

The increasing digitalization and virtualization of clinical care and the integration of services across providers call for an indicator of the resilience of the ecosystem of services that can be provided by the circles of care to individuals in a way that supports an active and independent living assisted by technology and with assistance when their condition periodically deteriorates. The ECM1 and ECM2 is an example of such ecosystem, which is supported by the blended physical and virtual services provided in the participant’s own home (ECM3 and ECM4) or referral to a bed outside the participant’s home (ECM5 or ECM6) in case of severe deterioration. The capacity to manage the individual in ECM1 and ECM2 relates to the competence of the health professionals to manage acute episodes remotely, their access to technology that supports their decisions, equipment in the participants’ homes that enables the handing of even more complex situations, and finally the participant’s and their relatives’ understanding of and ability to manage the fluctuations of their condition.

As our data points to not only the classification of COPD with respect to severity and type of exacerbations, the context and resources also need to be reported in comparable manners. Instead of comparing technology and capacity of all the involved persons, we suggest an index that describes the resilience of the ecosystem of health services provided virtually as an indicator of maturity and capacity to manage participants most efficiently with focus on their well-being.

We propose a Virtual Clinical Management Resilience Index (VCMRI) that reports on the success of keeping patients within the setting of the virtual clinical environment. The ratio should be calculated as the number of cases that can be managed in a virtual clinical environment divided by the total number of patients.

In the context of ECM, a VCMRI will be a ratio of the number of exacerbations that can be reversed at ECM2. We found 121 severe exacerbations among the participants in our study and by using the formula on VCMRI with ECM2 in the denominator, we calculated a VCMRI of 0.694 for ECM2 meaning that almost 70% of severe exacerbations mitigated in ECM2. This is of interest as all services in ECM2 are performed virtually without any physical contact to the surrounding health care system. The VCMRI may be an important tool for the development of future integrated systems as it can enable the documentation and comparison of the health care services’ ability to keep the patient at home, which is a service goal for most patients, who prefer receiving treatment and care in their own home [31,32]. Therefore, the VCMRI may contribute to the development of more integrated and personalized health care services fulfilling the patient’s wish to be at home.

With its model for describing activities, roles, contexts, and technologies, the ECM can be used to allocate resources both within the virtual ecosystem and in the physical activities from ECM3 to ECM6. In reporting the resilience index, the efforts and resources can be reported in accordance with the structure of the ECM with respect to roles of the actors, their activities, the technology involved, and geographical locations as well as roles and certification of the involved actors. Future studies reporting in this context may map themselves into this model and compare with our results.

### Strengths and Limitations

The strength of our study is the close contact to the participants in the period they are connected to the living lab. This provides our RCC staff with an insight into the everyday life of participants and how they manage their condition. The use of technology to document all the events for this study imposes a limitation as we cannot be entirely sure that all communications and actions are documented. Also, we do not have access to other health care IT systems at the individual level, which may result in an underestimation of hospital admissions relating to other conditions.

Another limitation is the recruitment of participants as it is a convenience sampling from the local community and without direct referral from hospitals or general practitioners. We may therefore miss those who were not able to use technology or were disengaged from the services where we recruited. With a more vigorous inclusion, we might be able to include more COPD patients, who have difficulties in managing their health. This could result in more severe courses including a higher rate of hospitalization as Wilkinson et al [8] demonstrated that those not seeking their GP or hospital specialists are in a higher risk of deterioration.

The observation period varied from 1 day to more than 2 years, and the lack of exclusion of the 4.6% who participated for less than a 14-day period may contribute to a lack of statistical differences between the risk factor groups with respect to the number of exacerbations and contact notes, as normalization of these data to patient year may have increased the variance of the data set. We have kept all the participants regardless of days spent in the study to conform to our research protocol, and we also find it important to keep these data as information about how early withdrawal contributes to the full picture of the population. It should be noted that all participants have been through 2 visits in their home by both a medical doctor and a member of the service team who introduced them to the equipment for self-tracking. Thus, withdrawal after only a few days signifies that despite of the enrolled participants being well informed, there is still a small group of participants who find it too difficult when left to self-monitoring. Unfortunately, for ethical reasons, we were not permitted to ask for a reason for the withdrawal.

Our study is an observational study without any controls. We are therefore not able to document the effect of the ECM intervention compared to how the participants would behave in relation to their contact pattern to conventional health services.

### Perspective

We recommend future studies focus on participants who have been enrolled at least 90 days prior to observations for 2 reasons. The first is to be able to obtain a steady state situation before activities and outcomes of the intervention are observed. The second is from a resource perspective, as our data indicate that the resources and efforts during enrollment and the first 30 days can only be justified if the COPD patients are using the services for a longer period, as their need for resources declines after 90 days (Figure 2).

Future studies should also be more focused on a more appropriate number of participants in each of the GOLD classes and risk factor groups, and they should also include matched controls or include a randomized control group.

### Conclusion

The establishment of a person- and health-centered and digitally supported environment to assist people living with COPD has offered us a unique opportunity to learn about the nature of COPD over a period of up to 2 years. We find a large variation in both to what extent the participants engage themselves in self-monitoring activities and their need for contact with health professionals. The participants who are in most need of interaction with the health professionals (both virtual and nonvirtual) and have with the highest number of exacerbations belong to risk factor group D. The participants’ interactions with the RCC staff were highest during the first month of enrollment as also seen in other studies, indicating that the participants develop their capability to manage their own condition over time. Our results are difficult to compare to other technology-assisted settings and we therefore suggest a resilience index, the VCMRI. Together with the ECM, the resilience index could be used as a scaffold to report on the success with mitigating participants at the virtual clinical management level and at the same time report on involved capacity. We also find a need for a classification of severe exacerbation which can translate the current GOLD recommendation of hospitalization as a criterion with resources and efforts comparable to these in a telehealth care setting including the practice of referral to hospitalization at home or community care.

## Abbreviations

COPD: chronic obstructive pulmonary disease
ECM: Epital Care Model
FEV_1_: forced expiratory volume during the first second
GOLD: Global Initiative on Chronic Obstructive Lung Disease
IT: information technology
PRO: patient-reported outcome
RCC: response and coordination center
VCMRI: Virtual Clinical Management Resilience Index
